# Simvastatin and Muscle: Zebrafish and Chicken Show that the Benefits are not Worth the Damage

**DOI:** 10.3389/fcell.2022.778901

**Published:** 2022-03-14

**Authors:** Laise M. Campos, Livia Guapyassu, Cyro Gomes, Victor Midlej, Marlene Benchimol, Claudia Mermelstein, Manoel Luis Costa

**Affiliations:** ^1^ Instituto de Ciências da Saúde, Universidade Federal da Bahia, Salvador, Brazil; ^2^ Instituto de Ciências Biomédicas, Universidade Federal do Rio de Janeiro, Rio de Janeiro, Brazil; ^3^ Laboratório de Ultraestrutura Celular, Instituto Oswaldo Cruz, Fundação Oswaldo Cruz, Rio de Janeiro, Brazil; ^4^ Centro Nacional de Biologia Estrutural e Bioimagem, Universidade Federal do Rio de Janeiro, Rio de Janeiro, Brazil; ^5^ Universidade do Grande Rio (UNIGRANRIO), Duque de Caxias, Rio de Janeiro, Brazil

**Keywords:** simvastatin, muscle, cholesterol, embryo, zebrafish

## Abstract

Simvastatin is one of the most common medicines prescribed to treat human hypercholesterolemia. Simvastatin acts through the inhibition of cholesterol synthesis. Unfortunately, simvastatin causes unwanted side effects on muscles, such as soreness, tiredness, or weakness. Therefore, to understand the mechanism of action of simvastatin, it is important to study its physiological and structural impacts on muscle in varied animal models. Here we report on the effects of simvastatin on two biological models: zebrafish embryos and chicken muscle culture. In the last years, our group and others showed that simvastatin treatment in zebrafish embryos reduces fish movements and induces major structural alterations in skeletal muscles. We also showed that simvastatin and membrane cholesterol depletion induce major changes in proliferation and differentiation of muscle cells in chick muscle cultures. Here, we review and discuss these observations considering reported data on the use of simvastatin as a potential therapy for Duchenne muscular dystrophy.

## Introduction

High cholesterol levels in the blood are associated with an increased risk of cardiovascular disease in humans. Therefore, statins have been the number one choice of physicians to control blood cholesterol levels since these drugs inhibit 3-hydroxy-3-methylglutaryl coenzyme A reductase (HMG-CoA), a key enzyme of the cholesterol biosynthetic pathway. Simvastatin effectively decreases blood cholesterol levels and the risk of heart problems in those at high risk. Unfortunately, simvastatin can induce myalgia, myopathy, and rhabdomyolysis (Nikolic et al., 2020; Turner and Pirmohamed, 2019). To further analyze the effects of statins in muscle at the cellular level, our group has been studying the effects of simvastatin treatment in zebrafish (*Danio rerio*) embryos. Zebrafish is a vertebrate biological model with several advantages, such as a well-characterized genome, easy of maintenance in laboratory conditions, and fast growth (Horzmann and Freeman, 2018).

## Materials and Methods

### Zebrafish Husbandry and Simvastatin Treatment

Adult, wild-type zebrafish (*Danio rerio*) were maintained in aquaria with recirculating water system at 28 ± 1°C on a 14:10 light/dark cycle in a vivarium localized at the Institute of Biomedical Sciences, Federal University of Rio de Janeiro (Rio de Janeiro, Brazil). Embryos were collected after breeding. Animals were handled and experimented according to Institutional Animal Care and Use Committee protocols under the number 039/20.

### Simvastatin Treatment

Embryos were dechorionated at 6 hpf and 11 hpf (hours post-fertilization) and placed in 100-mm Petri dishes filled with 50 ml of egg water (60 mg/L sea salts and 0.15% methylene blue). Embryos were treated with simvastatin (University Pharmacy, UFRJ) at low concentrations (from 3 to 6 nM) or higher concentrations (from 0.375 to 1 mM). Embryos were treated with simvastatin for 18 hs at 28°C until they completed 24 hpf and then were processed as necessary. Simvastatin was dissolved in ethanol (final concentration of 0.02%).

### Antibodies and Probes

Rabbit polyclonal antibody against desmin (code # D-8281) was from Sigma-Aldrich (United States). DNA-binding probe 4,6-Diamino-2-phenylindole dihydrochloride (DAPI) and Alexa Fluor 546-goat anti-rabbit IgG antibody was from Molecular Probes (United States).

### Immunofluorescence

Dechorionated zebrafish embryos were fixed in 4% paraformaldehyde in phosphate buffered saline (PBS) for 1 h at room temperature. Embryos were then permeabilized with 0.5% Triton-X 100 in PBS (PBS/T) three times for 30 min and incubated overnight at 4°C with primary antibodies (diluted 1:100 in PBS/T). Then, embryos were washed for 30 min with PBS/T and incubated for 1 h at 37°C with Alexa Fluor-conjugated secondary antibodies (diluted 1:200 in PBS/T). Nuclei were labeled with 0.1 μg/ml of DAPI in 0.9% NaCl. Embryos were mounted on #1.5 24 × 60-mm glass coverslips (with spacers) using Prolong Gold (Molecular Probes). Experiments were repeated four times.

### Image Acquisition and Processing

Zebrafish embryos were examined with brightfield microscopy in an Axiovert 100 microscope (Carl Zeiss, Germany) coupled to an Olympus DP71 high-resolution camera and with fluorescence microscopy in a DSU Spinning Disk confocal scanner mounted on an inverted fluorescent microscope (Olympus, Japan). Control experiments with only secondary antibodies showed only a faint background staining (data not shown). Image processing (brightness and contrast adjustments) was performed using Fiji software (Schindelin et al., 2012) and figure panels were produced with Adobe Photoshop software (Adobe Systems Inc., San Jose, CA, United States).

### Transmission Electron Microscopy

Dechorionated embryos were washed gently in warm PBS, pH 7.2, and fixed overnight in 2.5% glutaraldehyde, 4% formaldehyde and 5 mM CaCl_2_ in 0.1 M cacodylate buffer (pH 7.2), and were then post-fixed for 90 min in 1% OsO_4_ in 0.1 M cacodylate buffer containing 5 mM CaCl_2_ and 0.8% potassium ferricyanide. Embryos were then dehydrated in acetone and embedded in Epon. Ultra-thin sections were cut and stained with uranyl acetate and lead citrate, and analyzed using a JEOL 1210 transmission electron microscope (Jeol, Japan). Experiments were repeated four times.

## Results

### Zebrafish Embryo as a Robust Model to Study Structural Muscle Alterations After Simvastatin Treatment

Zebrafish is an ideal system to analyze somite development. It allows the study and comparison of different stages of muscle/somite development (anterior-posterior axis) within the same embryo (Stickney et al., 2000); while the anterior somites are older, the posterior somites are younger. Our lab has been exploring the optical transparency of the zebrafish embryo by using high-resolution fluorescence microscopy, which enables us to do a thorough structural analysis of the distribution of key muscle proteins during somite formation (Costa et al., 2002, 2008). After this characterization of normal myogenesis, we showed that simvastatin causes several alterations in zebrafish embryos, such as body shortening and bending, depending on the dose (Campos et al., 2015, 2016). In these papers, we reported that low concentrations (from 3 to 6 nM) had mild structural and important physiological changes, including decrease in intermediate filaments, myofibrils and adhesion structures, and reduction in heart beat and body movement. We also observed that higher concentrations (from 0.375 to 1 mM) caused extensive structural and physiological alterations, including almost complete absence of intermediate filaments, myofibrils, adhesion structures, heart beat and body movement (Campos et al., 2015, 2016). The dose range and accompanying alterations was about the same for embryos treated at 6 and 11 h post-fertilization (hpf) (Campos et al., 2015, 2016).

**FIGURE 1:**
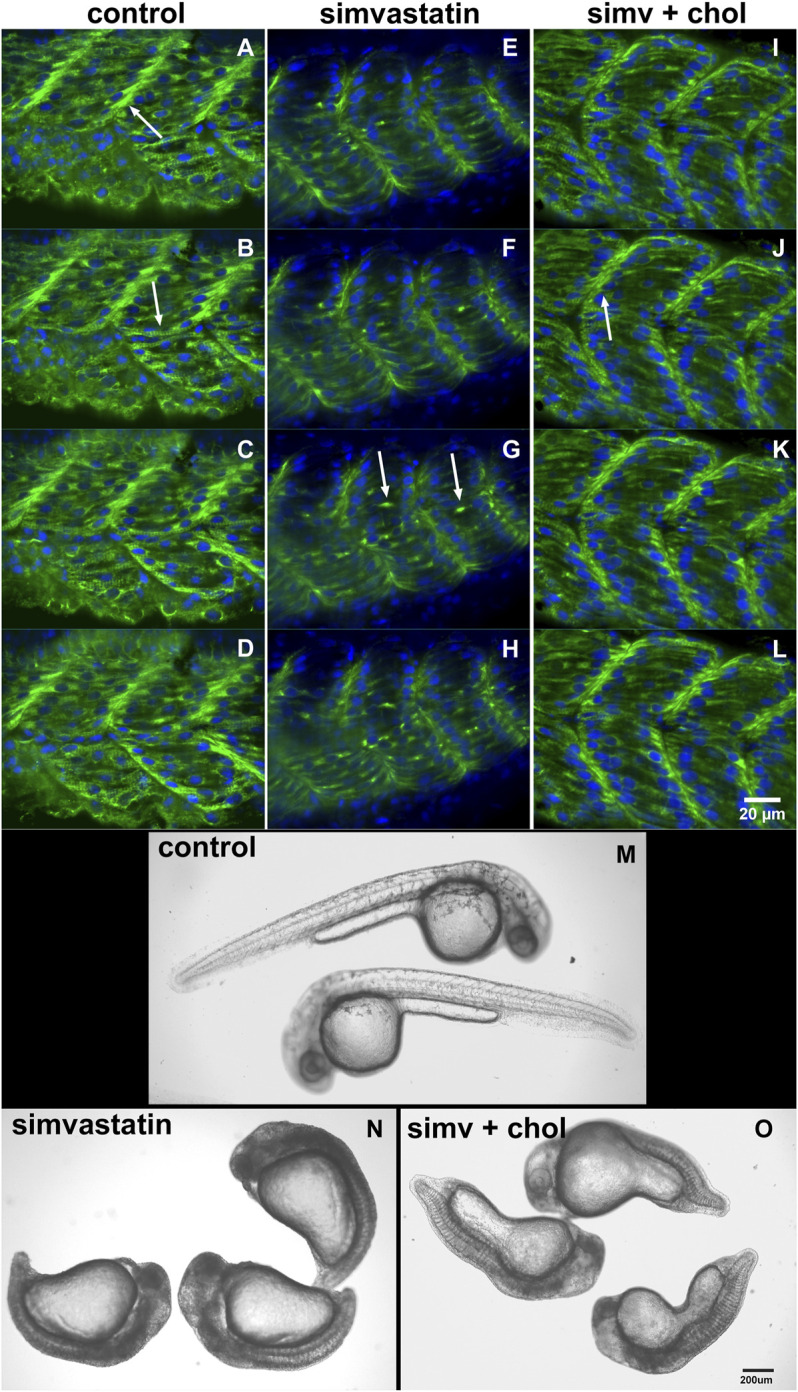
Cholesterol rescues the simvastatin-affected phenotype. The distribution of desmin (green) and nuclei (blue) in four consecutive focal planes of control **(A–D)**, 0.3 nM (low-dose) simvastatin-treated 48 hpf embryos **(E–H)**, and simvastatin combined with cholesterol **(I–L)** show that simvastatin causes important alterations that are rescued with cholesterol. Note that in control [non treated embryos, **(A–D)**] and in embryos treated with simvastatin together with cholesterol **(I–L)**, desmin concentrates in the septa between adjacent somites [arrows in **(A)** and **(J)**] and in sarcomeres along the somites [arrow in **(B)**]. Conversely, desmin accumulates in aggregates in somites [arrows in **(G)**] in simvastatin treated embryos **(E–H)**. In embryos treated with 0.75 μM simvastatin (high-dose), an important body compression is observed **(N)**, while 24 hpf control embryos have a long straight body **(M)**. Note that the addition of exogenous cholesterol can partially rescue the effects of simvastatin on body compression **(O)**. Scale bars: **(A–L)** = 20 μm, **(M–O)** = 200 μm.

**FIGURE 2:**
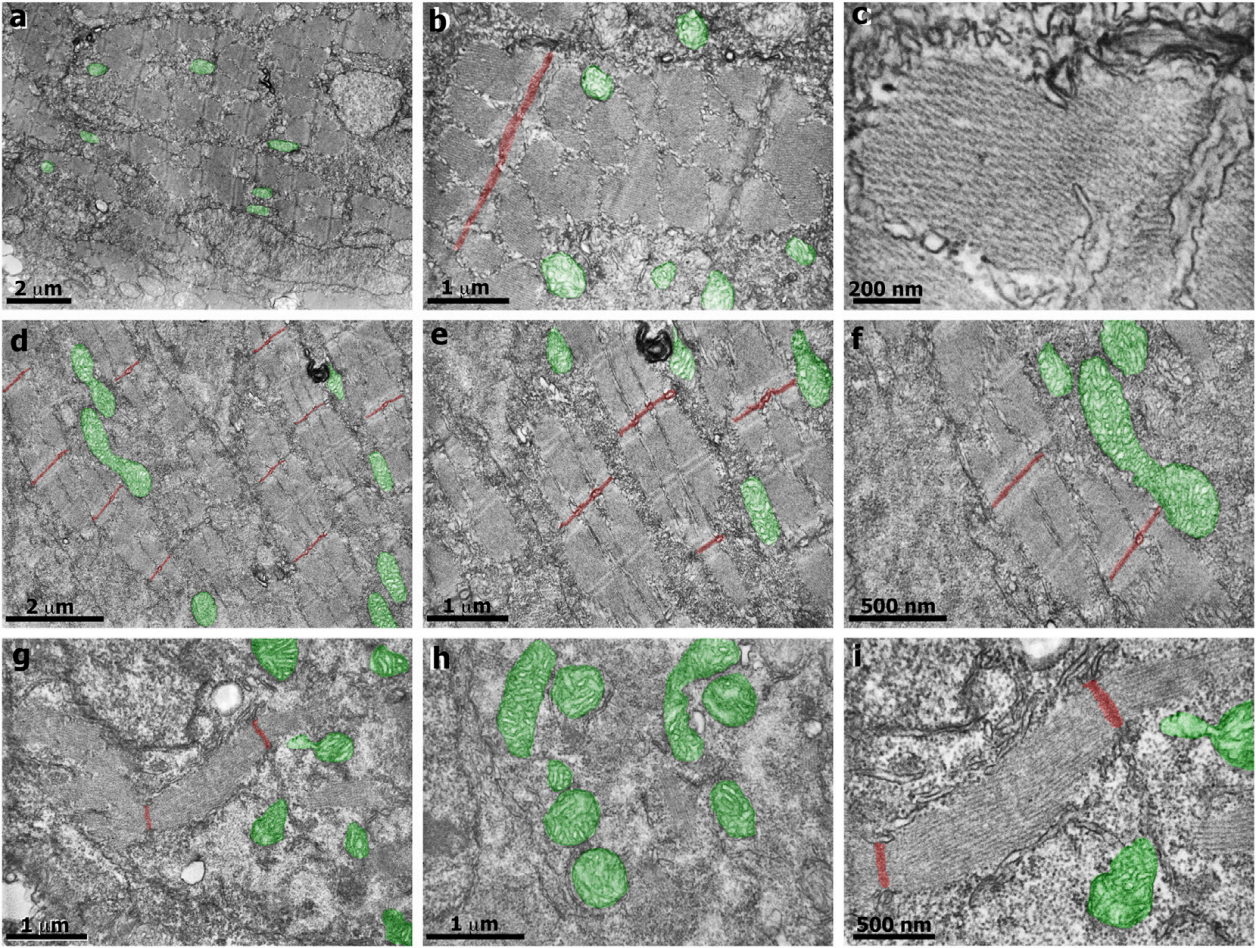
Transmission electron microscopy of zebrafish embryos treated with simvastatin. Control untreated zebrafish embryos **(A–C)** present organized bundles of myofibrils and exhibit well-preserved mitochondria (green). Note that the Z lines (red) are aligned and periodically spaced. The sarcomeric organization is better visualized in figure **(C)**. In the mild phenotype of simvastatin-treated embryos **(D–F)**, myofibrils are well-organized, and the alignment of Z lines is preserved. In the severe phenotype of simvastatin-treated embryos **(G–I)**, myofibrils are not aligned, vacuoles are observed **(G)**, as well as a higher number of mitochondria (green). Scale **(A,D)** 2 μm; **(B,E,G,H)** 1 μm; **(C)**, 200 nm; **(F,I)**, 500 nm.

In our earlier work we analyzed several aspects of the distribution of actin, desmin, vinculin, and laminin in simvastatin-treated embryos (Campos et al., 2015). To evaluate the specificity of simvastatin-induced alterations to cholesterol withdrawal, we treated embryos with simvastatin together with cholesterol, obtained from human blood in the form of low-density lipoprotein-LDL (Campos et al., 2015, 2016). Here we further explored the effects of simvastatin in zebrafish embryos. Using confocal microscopy analysis of the distribution of the muscle-specific protein desmin, it is possible to observe mild alterations in embryos treated with lower doses (3 nM) of simvastatin (Figure 1). While in control embryos desmin is concentrated in the septa (arrow in Figure 1A) and distributed in sarcomeres (arrow in Figure 1B), the protein is restricted to aggregates in simvastatin treated embryos (arrows in Figure 1G). The concomitant treatment of simvastatin and cholesterol preserved the concentration of desmin in the septa adhesion region (arrow in Figure 1J). We also observed structural alterations (extreme body shortening) in zebrafish embryos treated with high concentrations of simvastatin (Figures 1M,N), which could again be reverted to a certain degree by cholesterol (Figure 1O). While the whole-body development is affected with simvastatin treatment, we show using transmission electron microscopy that, compared to control (Figures 2A–C), there is extensive muscle-specific damage, particularly in the organization of sarcomeres and in mitochondria, both with low simvastatin doses (Figures 2D–F) and with high simvastatin doses (Figures 2G–I). Simvastatin induces alterations in myofibril alignment, vacuoles formation, and increased number of mitochondria. The effects of different doses of simvastatin treatment and/or treatment with simvastatin and exogenous cholesterol have not been previously described in the conditions we are showing here, and are in agreement with previous results of our group (Campos et al., 2015, 2016).

### Interfering With Cholesterol Availability in Chicken Muscle Cell Cultures

To address the mechanism of action and compare the impact of simvastatin in a different model, we also analyzed 24 h-chick primary myogenic cells treated with simvastatin (0.5 mM for 30 min). Our previous results showed a 20% decrease in cell number after simvastatin treatment (Portilho et al., 2012), in accordance with other studies in cultured human myoblasts (Van Vliet et al., 1996). It is interesting to point out that simvastatin, as we have shown before, reduces the number of cells compared to control zebrafish embryos (Campos et al., 2016). One possible mechanism of action of simvastatin is its effect on cell membrane stability. Cholesterol is a sterol lipid involved in the maintenance of plasma membrane fluidity and the formation and maintenance of lipid rafts. Lipid rafts are specialized membrane microdomains that contain high concentrations of cholesterol and glycosphingolipids and are involved in different cellular processes, such as signal transduction, endocytosis, and cell fusion and adhesion (Sezgin et al., 2017). Our lab has studied the consequences of membrane cholesterol depletion during chick myogenesis using primary cultures of embryonic chick muscle cells (Mermelstein et al., 2005; Mermelstein et al., 2007; Portilho et al., 2007; Costa et al., 2021). We showed that cholesterol depletion by MbCD (methyl-beta-cyclodextrin) induces an increase in the proliferation of myoblasts and the formation of myotubes (Portilho et al., 2012). These processes involve the release of Wnt3a molecules from chick muscle cells. The Wnt/beta-catenin pathway is involved in the proliferation of myoblasts, and we showed that cholesterol depletion by MbCD leads to an increase in the expression of cell cycle regulators and the master switch gene MyoD1 in chick myoblast cells (Portilho et al., 2012). The collection of these results supports the view that alterations in the amount of membrane cholesterol can lead to profound changes in muscle proliferation and differentiation (Possidonio et al., 2014). Curiously, the above-described data shows that MbCD and simvastatin have opposite effects in muscle cells: while cholesterol depletion (by MbCD) induces increased muscle cell proliferation, inhibition of cholesterol synthesis resulted in a reduction of muscle cell proliferation. These results point to a complex intracellular network of events associated with cholesterol in muscle cells and the importance of further investigations on the role of this lipid in muscle.

## Discussion

Simvastatin is used in treatment of human adult cholesteremia, and it is not recommend during pregnancy. Here we presented studies on the effects of simvastatin during myogenesis in zebrafish embryos and chick embryonic cultured cells. Therefore, a valid criticism of these models is that they are not equivalent to simvastatin effects in adult human muscle, and that adult tissues would be a better model. The advanges of using zebrafish embryos and cultured cells is that they are more susceptible to treatments and they allow a better visualization of muscle response at the molecular and cellular levels, which contribute to the understanding of mechanisms of action of drugs. Some of these processes, such as muscle proliferation and differentiation, also happen in adult muscle, during regeneration or in response to exercise. However, to correlate our current data with adult muscle, we are planning future experiments on the effects of simvastatin in adult zebrafish.

Our results show that simvastatin, depending on the dose, can induce major muscle alterations according to the work from several other groups using different statins and different animal or cell models. Hanai et al. (2007) reported that lovastatin promotes muscle fiber damage in zebrafish embryos and that atrogin-1 knockdown prevented these effects. These results suggest that atrogin-1 may be a critical mediator of the muscle damage induced by statins. Yu et al. (2009) showed that treatment of mouse C2C12 myotubes with simvastatin resulted in significant changes in gene expression, suggesting that alterations in the expression of some statin-regulated genes could be causative factors for statin toxicity in muscle. Wagner et al. (2011) used a small-molecule screening strategy to identify statin myopathy suppressors. They found one compound that attenuated the muscle side effects of statin toxicity, likely by influencing Rab prenylation. Huang et al. (2011) showed that the effects of statins on muscle were dependent on both the dosage and the duration of treatment. Piette et al. (2016) reported that a short-term statin treatment induced significant changes in the contractile profile of mice fast-twitch muscle EDL. In contrast, no effects were observed in the slow-twitch muscle Soleus. Furthermore, Pasha and Moon (2017) showed that statin-induced reduction in zebrafish larval response to tactile stimuli was reversed with coenzyme Q10, contributing to the understanding of the mechanisms of action of statin-induced myopathies.

The collection of our results shows that in zebrafish embryos, simvastatin treatment induces 1) a significant cholesterol reduction, 2) several dose-dependent alterations, 3) extensive changes in the distribution of microfilaments (actin), intermediate filaments (desmin), adhesion structures (vinculin) and extracellular matrix (laminin), 4) a reduction in cell number, and that 5) exogenous cholesterol is capable of recovering, at least in part, embryos treated with both low and high simvastatin doses. Furthermore, in chick muscle cultures, simvastatin induces a decrease in the number of cells while MbCD induces an increase in cell proliferation and muscle differentiation. These results provide a detailed characterization of the simvastatin-induced effects in muscles of zebrafish embryos and chicken muscle in culture. Since simvastatin reduces cell proliferation this may inhibit muscle regeneration. Future experiments are needed to study the possible consequences of simvastatin-induced reduction in cell proliferation during muscle regeneration. Also, it will be important in the future to analyse if these same effects occur when human muscle cells cultures are treated with simvastatin.

We suggest that these results should be considered in the discussion of the medical use of simvastatin, particularly in muscle degenerative diseases. Several studies were conducted in the last years aiming to understand the impact of simvastatin in the treatment of Duchenne muscular dystrophy (DMD), since statins have been shown to improve skeletal muscle health in ischemic limb diseases (Cowled et al., 2007; Köksoy et al., 2007; El-Azab et al., 2012). Whitehead and colleagues (2015) reported that simvastatin reduced plasma creatine kinase (CK) activity, muscle inflammation, and muscle damage while enhancing physiological function of skeletal muscle in dystrophic mdx mice. In contrast with these results, Mucha et al. (2021) and Verhaart et al. (2021) have recently shown that simvastatin does not alleviate muscle pathology in DMD mdx mice. Mucha et al. (2021) suggested that these conflicting results might be related to several divergences in methodology, including age and genetic background of the mdx mice, type and dose of the statin, route of administration, and length of time the drug was given to the animals. Whitehead and coworkers published a rebuttal letter (Whitehead et al., 2021) arguing that the conflicting results could be explained by the fact that neither Mucha et al. (2021) nor Verhaart et al. (2021) were able to achieve in their studies the therapeutic levels of plasma simvastatin, that Whitehead et al. (2015) attained. According to Whitehead and colleagues, low plasma levels of simvastatin are not sufficient to improve mdx muscle health and function. Aartsma-Rus et al. (2021) published another rebuttal corroborating that the differences in the result of the studies could be related to plasma levels of simvastatin. More studies are necessary to unravel the molecular and cellular mechanisms involved in statin-induced effects in different experimental models (animals and 3D muscle cell cultures) of degenerative muscle diseases, and zebrafish is among the most promising vertebrate models for these studies.

## Data Availability

The raw data supporting the conclusion of this article will be made available by the authors, without undue reservation.
